# Development of Sensitive Droplet Digital PCR Assays for Detecting Urinary *TERT* Promoter Mutations as Non-Invasive Biomarkers for Detection of Urothelial Cancer

**DOI:** 10.3390/cancers12123541

**Published:** 2020-11-27

**Authors:** Md Ismail Hosen, Nathalie Forey, Geoffroy Durand, Catherine Voegele, Selin Bilici, Patrice Hodonou Avogbe, Tiffany Myriam Delhomme, Matthieu Foll, Arnaud Manel, Emmanuel Vian, Sonia Meziani, Berengere De Tilly, Gilles Polo, Olesia Lole, Pauline Francois, Antoine Boureille, Eduard Pisarev, Andrei R. O. S. E. Salas, Sara Monteiro-Reis, Rui Henrique, Graham Byrnes, Carmen Jeronimo, Ghislaine Scelo, James D. McKay, Florence Le Calvez-Kelm, Maria Zvereva

**Affiliations:** 1 International Agency for Research on Cancer (IARC), 69372 Lyon, France; ismail.hosen@du.ac.bd (M.I.H.); ForeyN@iarc.fr (N.F.); geoffroy.durand@med.lu.se (G.D.); VoegeleC@iarc.fr (C.V.); biliciselin10@gmail.com (S.B.); patrice.avogbe@fast.uac.bj (P.H.A.); tiffany.delhomme@irbbarcelona.org (T.M.D.); FollM@iarc.fr (M.F.); sonia.meziani8@gmail.com (S.M.); olesia.lole@gmail.com (O.L.); pauline.francois@i2bc.paris-saclay.fr (P.F.); antoine.boureille@gmail.com (A.B.); andrei.escartinsalas@gmail.com (A.R.O.S.E.S.); ByrnesG@iarc.fr (G.B.); ghislaine.scelo@gmail.com (G.S.); MckayJ@iarc.fr (J.D.M.); 2 Department of Biochemistry and Molecular Biology, Faculty of Biological Sciences, University of Dhaka, Dhaka 1000, Bangladesh; 3 Le Creusot Hospital, 71200 Le Creusot, France; ArMANEL@hoteldieu-creusot.fr; 4 Department of Urology, Protestant Clinic of Lyon, 69300 Caluire-et-Cuire, France; emmanuel.vian@infirmerie-protestante.com (E.V.); berengeredetilly@hotmail.com (B.D.T.); gilles.polo@infirmerie-protestante.com (G.P.); 5 Faculty of Bioengineering and Bioinformatics, Lomonosov Moscow State University, 119234 Moscow, Russia; e.pisarev@fbb.msu.ru; 6 Santa Casa de Sao Paulo School of Medical Sciences, Sao Paulo 01221-020, Brazil; 7 Portuguese Oncology Institute of Porto, Research Center (CI-IPOP), 4200-072 Porto, Portugal; sara.raquel.reis@ipoporto.min-saude.pt (S.M.-R.); henrique@ipoporto.min-saude.pt (R.H.); carmenjeronimo@ipoporto.min-saude.pt (C.J.); 8 Department of Pathology, Portuguese Oncology Institute of Porto (IPOP), 4200-072 Porto, Portugal; 9 Institute of Biomedical Sciences Abel Salazar, University of Porto (ICBAS-UP), 4099-002 Porto, Portugal; 10 Department of Medical Sciences, University of Turin, 8-10124 Turin, Italy; 11 Department of Chemistry, Lomonosov Moscow State University, 119991 Moscow, Russia

**Keywords:** droplet digital PCR, liquid biopsy, bladder cancer, *TERT* promoter mutations, urinary biomarkers

## Abstract

**Simple Summary:**

The gold standard method for the diagnosis of bladder cancer (BC) is the invasive and expensive cystoscopy. Telomerase reverse transcriptase (*TERT*) promoter mutations occur frequently (60–90%) in BC. In this study, we developed highly sensitive droplet digital PCR (ddPCR) assays for detecting low-allelic fraction *TERT* promoter mutations (C228T, C228A, CC242-243TT and C250T) in urinary cell-free and/or cell pellet DNA of BC patients and compared their performance with our previously established NGS-based assay (UroMuTERT) in two independent case-control studies: DIAGURO (*n* = 89 cases and *n* = 92 controls) and IPO-PORTO (*n* = 49 cases and *n* = 50 controls). The sensitivity and specificity of the ddPCR assays in detecting *TERT* promoter mutations in BC cases and controls were very high and comparable to the UroMuTERT assay. However, the technical and analytical simplicity of the ddPCR assays make them suitable candidates for clinical implementation.

**Abstract:**

Somatic mutations in the *telomerase reverse transcriptase* (*TERT*) promoter regions are frequent events in urothelial cancer (UC) and their detection in urine (supernatant cell-free DNA or DNA from exfoliated cells) could serve as putative non-invasive biomarkers for UC detection and monitoring. However, detecting these tumor-borne mutations in urine requires highly sensitive methods, capable of measuring low-level mutations. In this study, we developed sensitive droplet digital PCR (ddPCR) assays for detecting *TERT* promoter mutations (C228T, C228A, CC242-243TT, and C250T). We tested the C228T and C250T ddPCR assays on all samples with sufficient quantity of urinary DNA (urine supernatant cell-free DNA (US cfDNA) or urine pellet cellular DNA (UP cellDNA)) from the DIAGURO (*n* = 89/93 cases and *n* = 92/94 controls) and from the IPO-PORTO (*n* = 49/50 cases and *n* = 50/50 controls) series that were previously screened with the UroMuTERT assay and compared the performance of the two approaches. In the DIAGURO series, the sensitivity and specificity of the ddPCR assays for detecting UC using either US cfDNA or UP cellDNA were 86.8% and 92.4%. The sensitivity was slightly higher than that of the UroMuTERT assay in the IPO-PORTO series (67.4% vs. 65.3%, respectively), but not in the DIAGURO series (86.8% vs. 90.7%). The specificity was 100% in the IPO-PORTO controls for both the UroMuTERT and ddPCR assays, whereas in the DIAGURO series, the specificity dropped for ddPCR (92.4% versus 95.6%). Overall, an almost perfect agreement between the two methods was observed for both US cfDNA (*n* = 164; kappa coefficient of 0.91) and UP cellDNA (*n* = 280; kappa coefficient of 0.94). In a large independent series of serial urine samples from DIAGURO follow-up BC cases (*n* = 394), the agreement between ddPCR and UroMuTERT was (i) strong (kappa coefficient of 0.87), regardless of urine DNA types (kappa coefficient 0.89 for US cfDNA and 0.85 for UP cellDNA), (ii) the highest for samples with mutant allelic fractions (MAFs) > 2% (kappa coefficient of 0.99) and (iii) only minimal for the samples with the lowest MAFs (< 0.5%; kappa coefficient 0.32). Altogether, our results indicate that the two methods (ddPCR and UroMuTERT) for detecting urinary *TERT* promoter mutations are comparable and that the discrepancies relate to the detection of low-allelic fraction mutations. The simplicity of the ddPCR assays makes them suitable for implementation in clinical settings.

## 1. Introduction

Bladder cancer (BC), accounting for 90% of urothelial cancer (UC), is the 10th most common cancer worldwide [1]. There are two major groups of BC patients with distinctive clinical outcome and molecular features: Non-muscle-invasive and muscle-invasive [2]. Owing to the high recurrence rate of the non-muscle-invasive BC and expensive cystoscopy as a gold-standard diagnostic method, BC is one of the most expensive cancer to treat and manage [3]. The current guidelines for routine diagnosis and monitoring of BC rely on a sensitive but invasive cystoscopy as the gold standard and urine cytology, which is relatively less sensitive than cystoscopy, especially for low-grade non-muscle-invasive BC [4]. The invasiveness, risk of complications, and high expense of the cystoscopy technique poses significant challenges to the management of BC, especially in subjects at sufficiently low-risk for BC, for whom invasive testing may be replaced by highly sensitive and specific methods for non-invasive detection of BC. Currently, clinical guidelines for the follow-up of patients with asymptomatic microscopic hematuria (AMH) recommend invasive cystoscopy testing, with only a small fraction of AMH patients developing bladder cancer [5]. Individuals exposed to BC carcinogens at their workplace and therefore under regular medical surveillance may also benefit from a BC screening strategy using urine biomarkers with higher diagnostic predictive values than the common non-invasive tests (hematuria and urine cytology). Urine-based biomarkers may therefore be useful in these clinical scenarios, thereby contributing to early detection of BC and subsequent monitoring [6]. However, to date, no reliable non-invasive biomarker has been proposed for routine BC clinical management or for screening populations at risk of BC. Therefore, detecting molecular alterations of the bladder tumor in the urine promises to be an important addition in the liquid biopsy of BC [7,8].

Two mutations in the promoter of the *Telomerase reverse transcriptase* gene (*TERT*), called C228T and C250T, are frequent in various human cancers and particularly in UCs (carcinomas of the bladder and upper urinary tract) where it is seen in 60–85% of cases (74% for non-invasive UCs) [9,10]. Should these mutations be detectable in the urine of BC patients as a means of liquid biopsy, they would provide an unprecedented opportunity for a simple diagnostic assay of UCs, which could be used for detection as well as monitoring progression and recurrence. We have recently developed a Next-Generation Sequencing (NGS)-based assay, called UroMuTERT, for the comprehensive detection of BC in urine cell pellet and cell-free DNA samples [11]. We specifically showed in case-control studies that urinary *TERT* promoter mutations have excellent sensitivity and specificity for the detection of BC [11] and that these mutations could also be detected in urinary DNA of asymptomatic individuals years prior to primary diagnosis of BC with high specificity [12], highlighting their potential to be used as simple and inexpensive non-invasive biomarkers for early detection of BC. While the NGS assay shows great promise in detecting *TERT* promoter mutations in urine samples, the complex laboratory workflow and requirement of extensive bioinformatics skills for data analysis are still considered as a bottleneck for their large-scale clinical application.

In this study, we report the development of sensitive droplet digital PCR assays for detecting *TERT* promoter mutations in urine samples. We also present for the first time the comparison of ddPCR- and NGS-based assays for the detection of *TERT* promoter mutations in urine samples from a series of BC patients and controls. 

## 2. Results

### 2.1. Optimization of the ddPCR Assays

**Figure 1:**
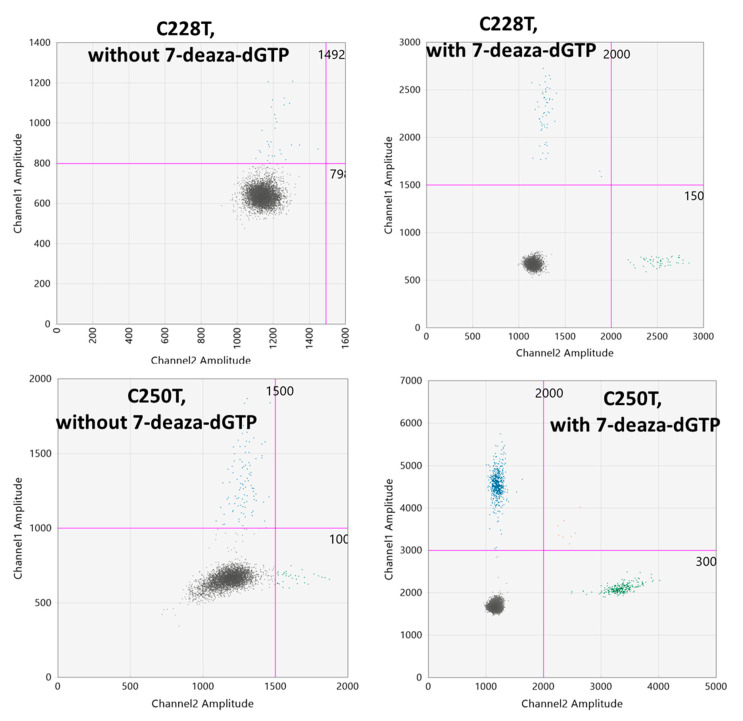
Effect of the 7-deaza-dGTP reagent (2 µM) in generating clear droplet clusters for C228T and C250T assays.

The GC content of the *TERT* promoter region is approximately 80% which causes the formation of G-quadruplex structures [13,14]. The two most common somatic mutations of the *TERT* promoter (C228T and C250T) occur within the terminal G-tracts of the second putative quadruplex sequence [14]. These higher-level organization at the *TERT* promoter mutation sites poses a significant threat for the ddPCR assays to show distinct droplet clusters. To resolve this issue, we used 7-deaza-dGTP, Li-salt at a 2 µM concentration. We optimized the concentration of 7-deaza-dGTP by a series of experiments with or without 7-deaza-dGTP in the PCR reactions (data not shown) and found that the addition of 2 µM 7-deaza-dGTP resulted in a clear separation of droplet clusters (Figure 1). The effect of 7-deaza-dGTP in improving the ddPCR cluster resolution is in concordance with the results obtained by Colebatch et al. [15].

### 2.2. Determination of the Threshold Number of Minimum Mutated Droplets to Call a Mutation

**Figure 2:**
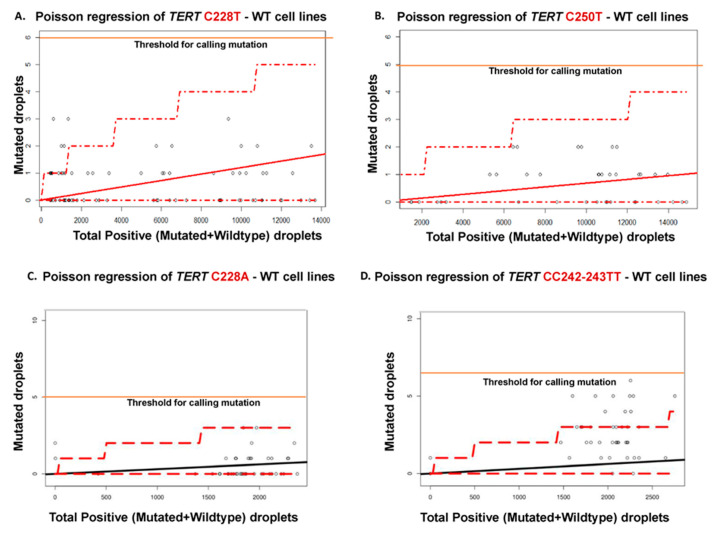
Determination of the threshold number of minimum droplets for calling a mutation. Poisson regression was generated from the number of droplets obtained from channel 1 (for mutated probe) and channel 2 (for wildtype probe) of 20 wildtype cell-lines for the C228T (**A**), C250T (**B**), C228A (**C**) and CC242-243TT (**D**) ddPCR assays. From this regression, the threshold for calling C228T, C250T, C228A and CC242-243TT mutations was set at 6, 5, 5 and 6 positive/mutated droplets, respectively.

In order to determine the false positive measures and consequently the threshold number of minimum mutated droplets detected in the channel with the mutated probe, 20 DNA samples wildtype for the respective *TERT* mutations were assayed using the respective ddPCR assays. We used the Poisson regression results generated for wild-type samples (Figure 2) to determine the level of unspecific fluorescence for each of the ddPCR assays and infer for each assay the minimum of positive droplets to consider a sample as harboring the mutation. A minimum number of 5 or 6 positive droplets (mutated blue droplets above the Channel 1 thresholds) were considered to be positive in ddPCR for C228A/C250T and C228T/CC242-243TT assays, respectively.

### 2.3. Determination of the Limit of Detection of the ddPCR Assays

The limit of detection (LOD) of the ddPCR assays was evaluated using serial dilutions of DNA from *TERT* C228T and C250T mutated cell lines in a background of DNA samples wild-type for these two mutations as depicted in Section 4. Quantities of 5 ng, 10 ng, 20 ng and 40 ng template DNA from these serially diluted samples were used for the ddPCR assays and the mutant allele fraction (MAF) was calculated as a fraction of the number of droplets detected in the channel-specific for the probe detecting the mutated allele to that of the wild-type allele. Our results show that the LOD of the ddPCR assays is a function of the amount of template DNA used as an input for ddPCR experiments Tables S1 and S2. The LOD (lowest detectable MAF without false positive) for the *TERT* C228T assay was 2.45%, 0.69%, and 0.18% for 10 ng, 20 ng and 40 ng of template DNA, respectively (Table S1). An exception was observed for 5 ng where the LOD was 0.6%, probably because of a relatively low number of total positive droplets. On the other hand, the LOD for the *TERT* C250T assay was 1.96%, 0.43%, 0.41% and 0.18% for 5 ng, 10 ng, 20 ng and 40 ng of template DNA, respectively (Table S2).

**Figure 3:**
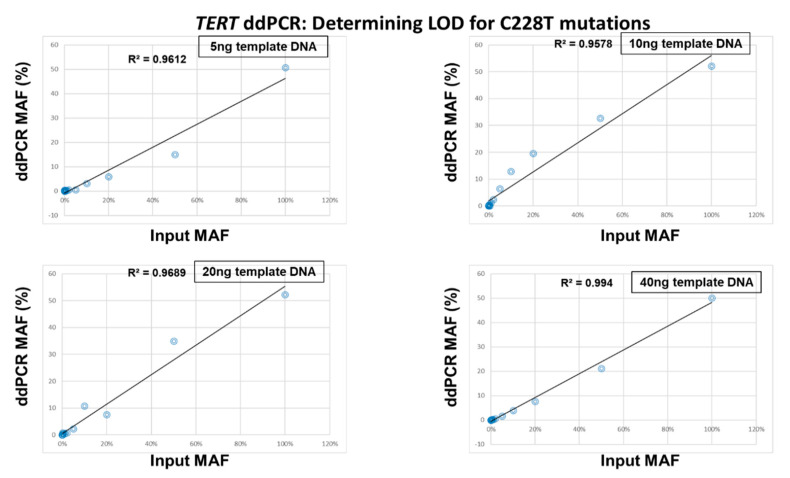
Linearity between the input Mutant Allelic Fractions (MAFs) and the experimentally determined MAFs for the *TERT* C228T assay. The theoretical percentage of MAFs from serially diluted C228T mutated template DNA into wild-type DNA was plotted against the experimentally determined ddPCR MAFs for 5 ng, 10 ng, 20 ng and 40 ng.

**Figure 4:**
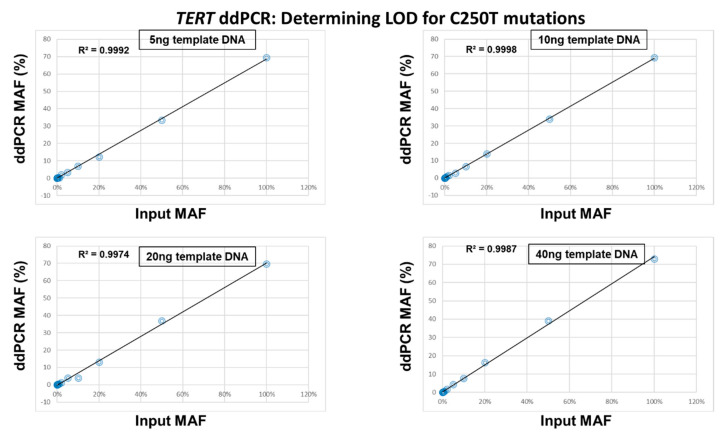
Linearity between the input Mutant Allelic Fractions (MAFs) and the experimentally determined MAFs for the *TERT* C250T assay. The theoretical percentage of the MAFs from serially diluted C250T mutated template DNA into wild-type DNA was plotted against the experimentally determined ddPCR MAFs for 5 ng, 10 ng, 20 ng and 40 ng.

We could also determine the quantitative linearity of MAFs using this experiment. For this purpose, the experimentally determined ddPCR MAFs were plotted against the MAFs theoretically present in the serially diluted input DNA (Figure 3 and Figure 4). The results showed that there was a strong correlation between the input MAFs and the MAFs calculated from the ddPCR experiments for both C228T and C250T assays and any amount of template DNA (R^2^ > 0.95) (Figure 3 and Figure 4).

### 2.4. Comparison of the ddPCR and UroMuTERT Assays for the Detection of TERT Promoter Mutations in Urinary and Tumor DNA

We performed the ddPCR assays for C228T and C250T on all the samples with a sufficient quantity of urinary DNA from the DIAGURO (*n* = 89/93 cases and *n* = 92/94 controls; US cfDNA or UP cellDNA) and the IPO-PORTO (*n* = 49/50 cases and *n* = 50/50 controls; UP cellDNA) series. All available tumors from the DIAGURO (*n* = 81) were also screened by ddPCR assays. Additionally, we validated the UroMuTERT NGS results for the C228A and CC242-243TT mutations in samples that were positive for these mutations.

In the DIAGURO series, the sensitivity and specificity of the ddPCR assays (C228T or C250T mutations) for detecting UC using either US cfDNA or UP DNA was 86.8% (95% CI 80.3–94.5) and 92.4% (95% CI 85.0–96.9), respectively (Table 1). Details about the availability of the samples or data related to the screening of US cfDNA and UP cellDNA individually are given in Table S3, together with related sensitivity and specificity. The overall sensitivity of the ddPCR assays (C228T or C250T mutations) in detecting primary UC cases in urine cellDNA from the IPO-PORTO cohort was 67.4% (95% CI 52.5–80.1) with a specificity of 100.0% (95% CI 92.9–100.0) (Table 1). We assessed the ddPCR analytical sensitivity of detecting urinary mutations in 81 BC cases with available matched tumors and identified urinary *TERT* promoter mutation(s) in US or UP in 69 of the 71 *TERT* mutated tumors (analytical sensitivity of 97.2%; 95% CI 90.2–99.7). Of the 10 tumors without *TERT* promoter mutations, seven were also negative in urine samples, three were positive for C228T in US or UP. In our previous study, we have established a single-gene assay based on the Ion Torrent Proton system (UroMuTERT) for the quantification of tumor-derived *TERT* promoter mutations load in urine cellDNA or cell-free DNA and evaluated its clinical performance in the DIAGURO and IPO-PORTO case-control studies [11]. Here, we compared the sensitivity, specificity, and accuracy of urinary *TERT* promoter mutations ddPCR assays for the detection of UC to that of UroMuTERT assay using the whole set of data (*n* = 187 in the DIAGURO and *n* = 100 in IPO-PORTO series) [11] and a restricted set of samples with available data for both techniques (*n* = 181 in the DIAGURO and *n* = 99 in IPO-PORTO series) (Table 1). We also compared the analytical sensitivity and specificity of detecting urinary *TERT* promoter mutations in corresponding matched tumors of DIAGURO cases (*n* = 81) using the two evaluated approaches.

Considering the latter condition, of the 32 IPO-PORTO BC cases detected with UroMuTERT, ddPCR assays confirmed the presence of the mutation in 30 cases and enabled the detection of two BC cases initially negative by UroMuTERT, suggesting a slightly increased sensitivity (67.4% with ddPCR vs. 65.3% with UroMuTERT). This was not observed in the DIAGURO series where, of the 80 BC cases detected with UroMuTERT, two were not confirmed by ddPCR assays, whereas one initial False-negative with UroMuTERT turned positive (sensitivity of 86.80% with ddPCR vs. 90.7% with UroMuTERT). Of note, the UroMuTERT sensitivity increased as compared to the previous published data involving 93 BC cases (87.1%) because three out of 12 BC cases without mutation after UroMuTERT could not be tested by ddPCR assays. The 100% specificity observed in the 50 IPO-PORTO controls after UroMuTERT held true with ddPCR assays, whereas of the 92 DIAGURO controls evaluated by ddPCR, seven false positives were identified with ddPCR and three by UroMuTERT (specificity of 92.4% versus 95.6%, respectively). Restricting the analysis to the 81 tumor DNA samples of the DIAGURO series screened by ddPCR did not change the UroMuTERT analytical sensitivity previously reported in 83 tumor DNA samples (analytical sensitivity of 98.6%; 95% CI 92.5–99.96) [11], which was comparable to the ddPCR analytical sensitivity (97.2%; 95% CI 90.2–99.7). The number of cases without *TERT* promoter mutations in tumors and with positive (*n* = 3) and negative (*n* = 7) urine tests remained unchanged between the two approaches. Regardless of the case and control status, we observed an almost perfect agreement between the two methods for both US cfDNA (*n* = 164; kappa coefficient of 0.91) and UP cellDNA (*n* = 280; kappa coefficient of 0.94) (Table S4). The almost perfect agreement held true for tumor DNA samples of the DIAGURO cases (*n* = 81; kappa coefficient of 0.95). Altogether, our results indicate that the two methods developed at our laboratory (ddPCR and UroMuTERT) for detecting urinary or tumor *TERT* promoter mutations are comparable and that the discrepancies relate to the detection of low-allelic fraction *TERT* promoter mutations (of 1% or less) in DNA in one of the two approaches.

### 2.5. Assessment of the Interrater Reliability of ddPCR and UroMuTERT Assays in Detecting Urinary TERT Promoter Mutations

While an almost perfect agreement between the two methods was shown in the two case-control studies, we further investigated whether it was maintained in a large series of serial urine samples from follow-up BC cases (*n* = 394) where we expected a fraction of samples to carry low MAF *TERT* promoter mutations. We screened US cfDNA and UP cellDNA samples for *TERT* promoter mutations with the two diagnostic approaches and calculated the kappa coefficients for all samples, for US cfDNA and UP cellDNA separately and we further stratified samples according to various level of MAFs obtained from the ddPCR data (Table 2). A strong agreement was also observed in this large series of urine samples (kappa coefficient of 0.85), regardless of urine DNA types (kappa coefficient of 0.89 for US cfDNA and of 0.85 for UP cellDNA). The highest kappa coefficient (0.99) was seen for samples with MAFs > 2% but the agreement between the two methods was considered as moderate for samples with MAFs < 2% (0.78) and for samples with MAFs < 1% (0.7). However, the agreement was minimal for the samples with the lowest MAFs (<0.5%; kappa coefficient 0.32) indicating that discrepancies between the mutational calls between the two methods are more likely to occur in samples with very-low MAFs, which confirms the observations made in the case-control studies (Table 2).

### 2.6. Correlation of MAFs between ddPCR and UroMuTERT Assays

**Figure 5:**
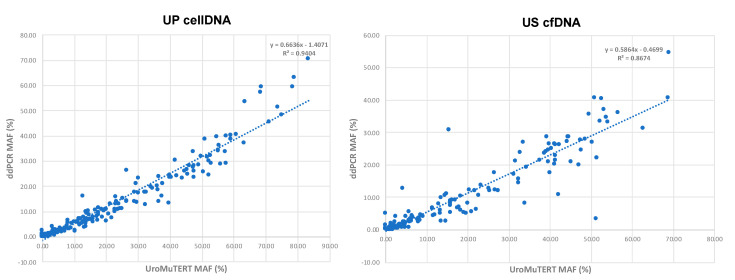
Correlation of *TERT* promoter Mutant Allelic Fractions (MAFs) between ddPCR and UroMuTERT assays in the two types of urinary DNA samples. Scatter plots of MAFs obtained by ddPCR and UroMuTERT assays run on US cfDNA (*n* = 172; left panel) and UP cellDNA (*n* = 222; right panel) from patients enrolled in the two case-control studies (DIAGURO and IPO-PORTO) and in the follow-up BC study.

Based on test results obtained from the two approaches on urine samples of the two case-control studies and on US cfDNA (*n* = 172) and UP cellDNA (*n* = 222) of the follow-up BC cases, we observed a strong correlation of urinary *TERT* promoter mutation MAFs for UP cell DNA (*r^2^* = 0.94) and US cfDNA (*r^2^* = 0.87), although the correlation was slightly less strong for the latter (Figure 5). For the same samples, the MAFs tended to be higher with the UroMuTERT assay than with the ddPCR assays.

Finally, in all evaluated urine samples from UC cases (including follow-up urine samples), the lowest *TERT* promoter MAF detected by ddPCR assays from 10 ng DNA were 0.25% and 0.1% in UP cellDNA and 0.17% and 0.14% in US cfDNA for C228T and C250T assays, respectively.

## 3. Discussion

Owing to the presence of exfoliated cells from the urinary tract epithelium in urine, the latter represents the best reservoir of urinary DNA for the analysis of tumor-derived mutations in BC patients. While liquid biopsy presents an opportunity for non-invasive detection and monitoring of bladder cancer, there are still technological bars to be crossed [16,17,18]. Due to the dilution of tumor-derived cells with normal epithelial cells in the urine of BC patients, the fractional abundance of the tumor cells can be very low, thereby requiring highly sensitive method(s) for the detection of these urinary mutations [18]. Droplet digital PCR (ddPCR) assays presents some advantages in detecting low-allelic fraction mutations in the urine [19]. The performance of ddPCR assays in detecting *TERT* promoter mutations in urine cfDNA has only been reported in a limited series of UC cases and controls [17] and more recently in UC cases undergoing monitoring of post-therapy disease [19]. Few other studies assessed ddPCR assays for detecting *TERT* promoter mutations but in different cancer types including melanoma and glioma and in a very small sample set [20,21,22].

In this study, we developed sensitive ddPCR assays for the detection of urinary *TERT* promoter mutations in UC and report comparable performance with our previously developed NGS-based UroMuTERT assay [11]. *TERT* promoter mutations are the most common somatic mutations in UC and their detection in urine has significant clinical applications [11,18,23]. A study by Kinde et al., has showed that the somatic mutations in the *TERT* promoter detected after the transurethral resection of the bladder tumor (TURBT) could be potential biomarkers of recurrent UC [10]. Another study has shown an increased risk associated with the detection of these mutations and recurrence of pT1 bladder cancer [24] and a more recent one demonstrated that the post-therapy urinary C228T status in BC cases under surveillance for relapse matched the clinical observations [19] thereby suggesting a plausible way of non-invasive dynamic follow-up of non-muscle invasive bladder cancer patients. In addition, within a prospective cohort study, urinary *TERT* promoter mutations were detected up to 10 years prior to clinical diagnosis of primary bladder cancer and were absent from matched controls [12]. All these studies provide exciting evidence for the utility of urinary *TERT* promoter mutations as non-invasive biomarkers for bladder cancer detection and monitoring.

Various NGS-based assays have been developed over time for the detection of *TERT* promoter mutations in urine [11,18]. However, due to the involvement of sophisticated laboratory workflows, extensive bioinformatics post-processing and analysis of the data, the NGS-based assays are not broadly implemented in the clinic [25]. On the other hand, the advantages of the ddPCR assays are its superior sensitivity, fast processing time, independence from extensive bioinformatics post-processing, and analysis of the data [26,27]. In our study, the LOD tests using different amounts of serially diluted cell line template DNA showed that our assays could detect as low as 0.18% for the C250T and the C228T mutant DNA. Our results suggest that the LOD for these mutations using ddPCR assays is also influenced by the amount of input template DNA. The LOD proved to be lower when the amount of template DNA was increased from 5 ng to 40 ng DNA, confirming the findings of a recent study [28]. This LOD is slightly better than that of our previously developed UroMuTERT assay for which the limits of detection were 0.5% and 0.8% for the C250T and C228T mutations, respectively [11]. A recent study on ddPCR assays for detecting *TERT* promoter mutations in melanoma has shown a similar LOD using *TERT* mutated melanoma cell lines [22]. When applied to urine samples of UC cases, the lowest *TERT* promoter MAF detected by ddPCR assays from 10 ng US cfDNA (*n* = 172) or UP cellDNA (*n* = 222) were equivalent to the LOD measured with cell-lines, demonstrating the transferability of the thresholds to such sample types. While the LOD of standard ddPCR assays for other common somatic mutations such as BRAF V600E could be as low as 0.001% [29], the high GC content and repetitive sequences of the *TERT* promoter region makes it more difficult to get a clear separation of mutated droplets against the background noise [30].

We also observed that, for the same samples, the MAFs tended to be higher with the UroMuTERT assay than with the ddPCR assays. We postulated that the first PCR-based step of the UroMuTERT assay may preferentially amplify the mutated *TERT* allele as a result of incomplete denaturation of extremely high GC-rich heteroduplex fragments, thus resulting in higher MAFs than the ones observed with ddPCR assays, which is based on absolute quantification of the target and should therefore represent a more precise measurement of the MAFs than the ones measured by UroMuTERT. Furthermore, the urinary sample type may matter when comparing the MAF between the two methods and may explain the difference in the R^2^ for US cfDNA and UP cellDNA. Few more outliers of the regression were observed for US cfDNA than for UP cellDNA. This may be explained by the differences in the distribution of US mutated and wild-type cfDNA size profiles between samples and the differences in amplicon size between UroMuTERT (147bp) and ddPCR (64bp for C228T/A and 88bp for C250T and CC242-243TT) assays; the latter favoring the amplification of smaller thus highly degraded urinary cfDNA forms, which have been shown to be predominant in such sample [31]. Altogether, this may partly explain the minor discrepancies of low-level urinary *TERT* promoter mutations observed between the two methods. Indeed, in the urine pellet DNA (UP DNA) from the BC cases in the IPO-PORTO series, the ddPCR assays failed to replicate positive mutations calls for two samples analyzed with the UroMuTERT assay, but enabled the mutation detection in two additional BC cases initially negative with the UroMuTERT assay, keeping the overall sensitivity to the similar levels. For the DIAGURO series, the sensitivity of the ddPCR assays was slightly lower (by around 3%) compared to the UroMuTERT assays. However, this decreased sensitivity of the ddPCR assays could be due to the fact that three out of 12 BC cases without mutation after UroMuTERT could not be tested by ddPCR assays. While the 100% specificity observed in the IPO-PORTO series was found for both the UroMuTERT and ddPCR assays, the specificity of the ddPCR assays in the DIAGURO controls was lowered compared to UroMuTERT (specificity of 92.4% versus 95.6%, respectively). While applying a more strict interpretation of the Cohen’s kappa coefficient developed by McHugh to better reflect the level of agreement between two methods in health-related studies [32], an almost perfect agreement was still observed between ddPCR assays an UroMuTERT for both US cfDNA (*n* = 164; kappa coefficient of 0.91) and UP cellDNA (*n* = 280; kappa coefficient of 0.94). This was confirmed in a large independent series of serial urine samples from follow-up BC cases, in which, when stratified by MAF, the agreement remains almost perfect for samples with MAFs > 2% (kappa coefficient of 0.99) but dropped to moderate for samples with MAF < 1% and to minimal for the samples with the lowest MAFs (< 0.5%), indicating higher rates of false positive or false negative calls in samples with very-low MAFs. Finally, there was also an almost perfect agreement between UroMuTERT and ddPCR assays for the detection of the mutations in the tumors, further attesting to the robustness of the two methods. In addition, we observed a high concordance of mutational status between tumors and matched urine samples in DIAGURO BC cases with analytical sensitivities of 98.6% for UroMuTERT and 97.2% for ddPCR assays and therefore only a small fraction of false negatives, likely reflecting in such cases the existence of minor tumoral *TERT* mutated clones (MAF < 1%) undetectable in urine samples. Bladder tumors are characterized by intra- and inter- tumoral heterogeneity both at the molecular and cellular levels [33,34]. The co-existence of multiple sub-clonal populations, including multiple *TERT* mutated clones has been also reported in bladder tumors [11,24]. As urine represents of ‘screenshot’ of the bladder tumor heterogeneity, tumor-derived mutations may be detected in urine that would not necessarily be captured by a unique piece of biopsy due to the heterogenous nature of this cancer type. This was observed in three out of ten DIAGURO cases with wild-type biopsy, highlighting the superiority of urine over biopsy in reflecting mutational burden of heterogenous bladder tumors. This observation further supports the importance of implementing testing for urinary *TERT* promoter mutations for the detection of BC with well validated methodologies.

In conclusion, the two methods developed at our laboratory (ddPCR and UroMuTERT) for detecting urinary *TERT* promoter mutations are comparable. The high sensitivity and specificity of the ddPCR urinary *TERT* promoter mutations assays in the two independent cohorts investigated in this study provides evidence that these tests could be easily implemented into the clinic for the non-invasive detection of bladder cancer.

## 4. Materials and Methods

### 4.1. Study Population, Sample Collection and Processing

The urine and tumor samples used in this study were from participants who were recruited as a part of two separate case-control studies: the DIAGURO and IPO-PORTO case-control series. Both of the studies were approved by the relevant ethical review committees (IARC Ethics Committee, ethical approvals No. 15-23 and No. 15-23-A1) and written informed consent was obtained for all participants. The details of these two case-control series are described elsewhere [11]. Briefly, the DIAGURO case-control study included patients with post-surgery histological confirmation of primary or recurrent UC (BC or/and Upper tract urothelial cell carcinoma (UTUCC) at any stage and grade) as cases (*n* = 93) and patients with urological pathological conditions other than UC as controls (*n* = 94). Tumor-urine-blood trios for cases and urine-blood duos for controls were collected prospectively in appropriate collection tubes. The blood and urine samples were processed within two hours of collection and DNA from plasmas, white blood cells (WBC), urine supernatants (US), urine pellets (UP), and tumor tissues were extracted using standard protocols [11]. Histological review of the tumor tissues was performed by a qualified pathologist. The IPO-PORTO case-control replicative series included cellDNA from urine pellets of 50 primary BC cases and 50 healthy controls with no history of cancer. The samples were part of the Biobank of the Portuguese Oncology Institute of Porto. The follow-up of the DIAGURO cases with non-muscle invasive UC is currently being organized for 56 patients with a collection of urine samples (US and UP) at each medical examination during medical surveillance for recurrence. Samples were processed and stored as described in the previous study and at the time of the present study, 394 urine samples were screened with UroMuTERT following the same protocol as previously described [11] and with ddPCR as described below.

### 4.2. Detection of TERT Promoter Mutations Using ddPCR Assay

We have developed and validated droplet digital PCR (ddPCR) assays for all the four *TERT* promoter somatic mutations found in our previous study using the UroMuTERT assay as previously described [11]. The targeted mutations include the most prevalent C228T and C250T, as well as the rare C228A, CC242-243TT mutations (Table S5, Figure S1).

For each ddPCR assay, 5′-FAM or 5′-HEX reporter dye and 3’ Iowa Black Fluorescent quencher were designed (Biorad, Hercules, California, USA). We selected positive samples with known *TERT* promoter mutations and tested our experimental conditions. A 22 µL reaction mix was prepared using 10 ng of DNA was used as a template, 11 µL of 2x ddPCR supermix-no dUTP (Biorad), 1.1 µL of 20× FAM and HEX probes for mutated and wildtype alleles, 1.1 µL of RsaI restriction enzyme (10 U/µL) and 0.2 µL of 7-deaza-dGTP, Li-salt (2 µM). The droplets were generated using the autoDG droplet generator (Biorad). The PCR amplifications of the droplets were carried out separately for the C228T, C228A, CC242-243TT and C250T assays using the following PCR conditions: 95 °C for 10 min, 40 cycles of 94 °C for 30 s, ramp 2.5/s, 54 °C for C228T assay (55 °C for the C228A and CC242-243TT, 64 °C for C250T assay; Figures S2 and S3) for 30 s followed by 98 °C for 10 min and then kept at hold at 12 °C. The fluorescent intensity of each droplet was measured using the droplet reader QX200 (Biorad). Analysis of ddPCR data was performed using QuantaSoft^TM^ Analysis Pro1.0.596 software (Biorad). The preparation of the droplets using the AutoDG, the subsequent PCR amplification, and the measurement of the fluorescent intensity using the QX200 droplet reader were performed in three separate rooms specific for these respective purposes to ensure a contamination-free environment. The 2D amplitude plots from the QuantaSoft^TM^ analysis pro software were analyzed by setting the threshold amplitudes for both the mutated and wild type channels. The thresholds for the channel 1 (mutated probe) for the C228A, C228T, CC242-243TT and C250T probes were 700, 1500, 500 and 3500, respectively. For the channel 2 (wild-type probe) the thresholds for C228A, C228T, CC242-243TT, and C250T probes were 1250, 1750, 1440, and 2000, respectively (Figure 1).

**Figure 6:**
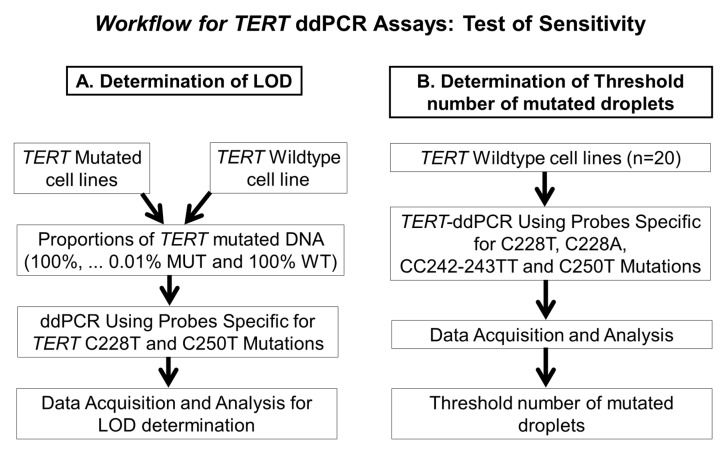
Schematic representation of the strategies for determining (**A**) the limit of detection (LOD) and (**B**) the threshold number of mutated droplets to call a mutation.

Two important technical aspects of developing ddPCR assays are the determination of the threshold number of droplets in the channel detecting the probe for the mutant allele and the limit of detection of the assays. Figure 6 explains the strategy for addressing these two points.

### 4.3. Determination of the False-Positive Measures and Threshold Number of Minimum Mutated Droplets to Call a Mutation

**Figure 7:**
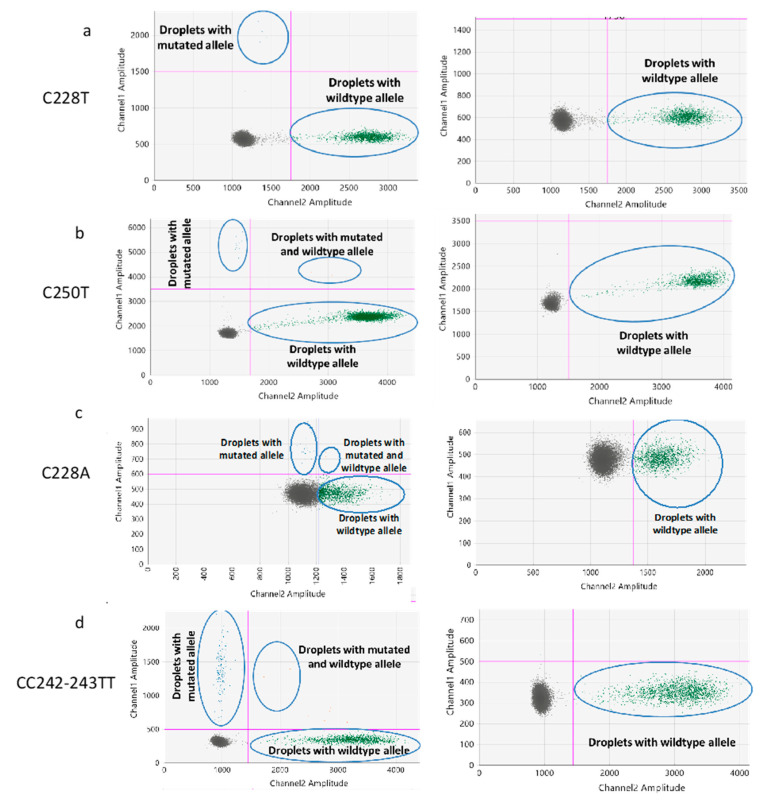
Examples of 2D scatterplots obtained from *TERT* promoter mutation ddPCR assays in representative samples. Assays testing for C228T (**a**), C250T (**b**), C228A (**c**), and CC242-243TT (**d**) mutations are displayed in four examples of mutated samples (left panels) and in four examples of wild-type samples (right panels). In the left panel, fluorescent probes (FAM) detect respective mutations as exemplified by the count of droplets with mutated alleles, while in the right panel, wild- type samples do not show any positive droplets (FAM) above the threshold line but show droplets with HEX fluorescence associated with wild-type probes. The pink lines are the thresholds for channel 1 (mutated probe) and channel 2 (wild-type probe) for the ddPCR mutation assays.

As recommended by the Clinical and Laboratory Standards Institute (CLSI) guidelines, negative samples should be measured to assess the probability distribution function of false-positive events detected in negative control samples [35]. With regards to ddPCR assays, one important technical aspect is to set up, for each assay, the threshold number of droplets in the channel detecting the probe for the mutant allele for calling a mutation beyond the background number of false-positive droplets. In order to determine the threshold number of positive droplets for calling a mutation, the ddPCR assays for the C228T, C228A, CC242-243TT and C250T mutations were performed on a series of cell lines which are wild-type at these genomic positions. The 2D amplitude plots from the QuantaSoft^TM^ analysis pro software were analyzed by setting the threshold amplitudes for both the mutated and wild type channels. The defined signal thresholds for the channels 1 and 2 for the C228A, C228T, CC242-243TT and C250T probes were used (Figure 7). Then Poisson regression of the number of droplets above the threshold was generated from the count of channel 1 (for mutated probe) and of channel 2 (for wildtype probe).

### 4.4. Determination of the Limit of Detection of the ddPCR Assays

Since the ddPCR assays are capable of detecting very low allelic-fraction mutations, another important technical development was to evaluate the limit of detection (LOD) of the *TERT*-ddPCR assays. To determine the LOD of the C228T and C250T assays, cell lines with characterized C228T and C250T *TERT* promoter mutations (HepG2 and A375, respectively) were used. Briefly, the genomic DNA from the HepG2 and A375 cell lines were serially diluted into *TERT*-wildtype DNA obtained from white blood cells of healthy controls to achieve from 100% cell lines DNA to 0% mutant allele fractions. Each of the serially diluted samples was tested using the respective ddPCR assays using the conditions mentioned above.

### 4.5. Comparison of the ddPCR and UroMuTERT Assays for the Detection of TERT Promoter Mutations in the Urine Samples of UC Cases and Controls

**Figure 8:**
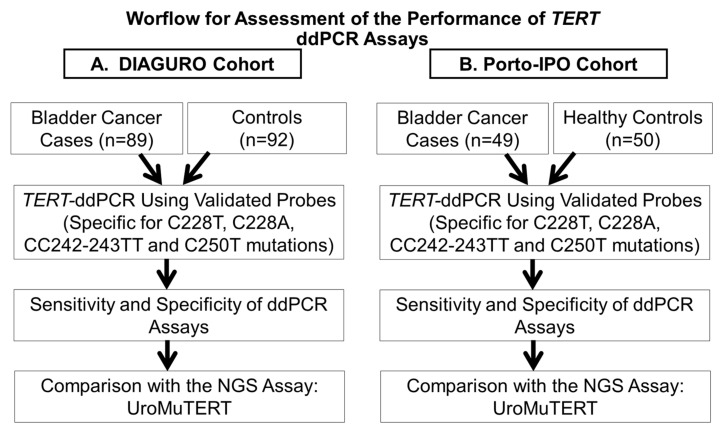
Schematic representation of the workflow for assessing the performance of the ddPCR assays for detecting *TERT* promoter mutations in urine of bladder cancer patients and controls in two independent cohorts.

All the samples from the DIAGURO and IPO-PORTO series with a sufficient amount of urinary DNA after the UroMuTERT assay was previously performed, were analyzed using the ddPCR assays and the results were compared to those of the UroMuTERT assay [11]. For this, we used 10 ng of urinary DNA (urine pellet or urine supernatant) as a template in a 22 µL reaction as described above (Figure 8). The fluorescent intensity of each droplet was measured using the droplet reader QX200 (Biorad). Analysis of ddPCR data was performed using QuantaSoft^TM^ Analysis Pro1.0.596 software (Biorad). All laboratory analyses were conducted blindly to the case or control status of the samples.

### 4.6. Statistical Analysis

Clinical sensitivity, specificity, and accuracy of the urinary *TERT* promoter mutations were calculated for the detection of BC using UroMuTERT or ddPCR assays and their confidence intervals computed using the Clopper-Pearson method [36]. True positives were defined as BC cases with a positive *TERT* urine test (ddPCR or UroMuTERT); False positives as subjects with a positive *TERT* urine test and no evidence of BC; False negative as BC cases with a negative *TERT* urine test and True negatives as subjects with a *TERT* negative test and no evidence of BC. The agreement between ddPCR and UroMuTERT assays was evaluated using Cohen’s kappa index which indicates the proportion of agreement beyond that expected by chance [37]. Cohen suggested interpretation to categorize the kappa index, where < 0.00 is a poor agreement, 0–0.2 is a slight agreement, 0.21–0.40 is a fair agreement, 0.41–0.60 is a moderate agreement, 0.61–0.80 is a substantial agreement, and 0.81–1.00 is an almost perfect agreement has been suggested to be too lenient for health-related studies [32]. We, therefore, used McHugh’s interpretation of the Cohen’s kappa index to infer the level of agreement between the two tested methods for mutations detection, where 0–0.2 is none agreement, 0.21–0.39 is a minimal agreement, 0.40–0.59 is a weak agreement, 0.60–0.79 is a moderate agreement, 0.80–0.90 is a strong agreement and > 0.9 is an almost perfect agreement [32].

## 5. Conclusions

Both ddPCR-based and NGS-based (UroMuTERT) assays established for the detection of urinary *TERT* promoter mutations demonstrated good and comparable performance for the non-invasive detection of urothelial cancer. Interrater reliability analysis showed a strong agreement between the two methods and an almost perfect agreement for mutant allelic fractions above 2%. Some discrepancies are however observed for the detection of low-allelic fraction mutations. The technical and analytical simplicity of ddPCR assays over the NGS-based assays could ease the future clinical implementation of the non-invasive tests for the detection and monitoring of urothelial cancer.

## Figures and Tables

**Table 1:** Performance of urinary TERT promoter mutations in detecting UC by ddPCR and UroMuTERT Assays.

C228T or C250T Mutations	ddPCR Assays		UroMuTERT Assay			
	DIAGURO Cohort	PORTO Cohort	DIAGURO Cohort a	PORTO Cohort a	DIAGURO Cohort b	PORTO Cohort b
	US cfDNA or UP DNA	UP DNA	US cfDNA or UP DNA	UP DNA	US cfDNA or UP DNA	UP DNA
Total Number analyzed	(*n* = 181)	(*n* = 99)	(*n* = 181)	(*n* = 99)	(*n* = 187)	(*n* = 100)
True Positive-no	79	33	80	32	81	33
True Negative-no	85	50	88	50	89	50
False Positive-no	7	0	4	0	5	0
False Negative-no	10	16	9	17	12	17
Sensitivity (95% CI)-%	86.8 (80.3–94.5)	67.4 (52.5–80.1)	90.7 (83.1–95.7)	65.3 (50.4–78.3)	87.1 (78.6–93.2)	66.0 (51.2–78.8)
Specificity (95% CI)-%	92.4 (85.0–96.9)	100.0 (92.9–100.0)	95.6 (89.2–98.8)	100.0 (92.9–100.0)	94.7 (88.0–98.3)	100.0 (92.9–100.0)
Accuracy (95% CI)-%	91.3 (86.2–95.0)	90.2 (82.6–95.3)	93.1 (88.5–96.3)	89.6 (81.8–94.8)	92.4 (90.6–94.0)	89.8 (87.8–91.6)

a: Data from Avogbe et al. [11], with the samples screened with both ddPCR and UroMuTERT assays. Samples with not enough DNA or ddPCR data were excluded for a precise comparison of the performance of the two methods (*n* = 6 from the DIAGURO series and *n* = 1 from the IPO-PORTO cohort). b: Original data from Avogbe et al. [11].

**Table 2:** Interrater reliability of ddPCR and UroMuTERT assays in detecting urinary TERT promoter mutations in a large series of serial urine samples from follow-up BC cases.

Sample Type	UroMuTERT-MUT		UroMuTERT-WT		Kappa Coefficient
	ddPCR-MUT	ddPCR-WT	ddPCR-MUT	ddPCR-WT	
	*n*	*n*	*n*	*n*	
All Follow-up cases (UP cellDNA and US cfDNA)	173	10	15	196	0.87
All US cfDNA Follow-up cases	86	3	9	123	0.89
All UP cellDNA Follow-up cases	87	7	6	73	0.85
Cases US/UP follow-up with MAF < 2% ddPCR	64	10	14	73	0.78
Cases US/UP follow-up with MAF > 2% ddPCR	109	0	1	123	0.99
Cases US/UP follow-up with MAF < 1% ddPCR	18	7	4	73	0.70
Cases US/UP follow-up with MAF > 1% ddPCR	155	3	11	123	0.90
Cases US/UP follow-up with MAF < 0.5% ddPCR	3	7	3	73	0.32
Cases US/UP follow-up with MAF > 0.5% ddPCR	170	3	12	123	0.90

US: Urine Supernatant. UP: Urine Pellet. MAF: Mutant Allelic Fraction.

## Supplementary Materials

The following are available online at https://www.mdpi.com/2072-6694/12/12/3541/s1, Figure S1: The TERT core promoter region with the primer and probe sets shown for each of the assays, Figure S2: 1D amplitude plot for Channel 1 (respective to mutated probe) and Channel2 (respective for wildtype probe) for the C228T assay at different annealing temperatures, Figure S3: 1D amplitude plot for Channel1 (respective to mutated probe) and Channel2 (respective for wildtype probe) for the C250T assay at different annealing temperatures, Table S1: Determination of Limit of Detection for the C228T mutations using cell line DNA, Table S2: Determination of Limit of Detection for the C250T mutations using cell line DNA, Table S3: Performance of urinary TERT promoter mutations in detecting UC by ddPCR based method, Table S4: Concordance of TERT promoter mutations status in urinary DNA of the DIAGURO and IPO-PORTO case-control studies Table S5: Probes for detecting C228T and C250T mutations in the ddPCR assay.
