# Supplementary material for: Development of Sensitive Droplet Digital PCR Assays for Detecting Urinary TERT Promoter Mutations as Non-Invasive Biomarkers for Detection of Urothelial Cancer
Source: Cancers (Basel). 2020 Nov 27;12(12):3541. doi: 10.3390/cancers12123541 (PMC7761513; doi:10.3390/cancers12123541)
Supplement: Supplementary file 1 [file cancers-12-03541-s001.pdf]

Supplementary Material: Development of Sensitive Droplet Digital PCR Assays for Detecting Urinary TERT Promoter Mutations as Non-invasive Biomarkers for Detection of Urothelial Cancer

Md Ismail Hosen, Nathalie Forey, Geoffroy Durand, Catherine Voegele, Selin Bilici, Patrice Hodonou Avogbe, Tiffany Myriam Delhomme, Matthieu Foll, Arnaud Manel, Emmanuel Vian, Sonia Meziani, Berengere De Tilly, Gilles Polo, Olesia Lole, Pauline Francois, Antoine Boureille, Eduard Pisarev, Andrei R.O.S.E. Salas, Sara Monteiro-Reis, Rui Henrique, Graham Byrnes, Carmen Jeronimo, Ghislaine Scelo, James D. McKay, Florence Le Calvez-Kelm and Maria Zvereva

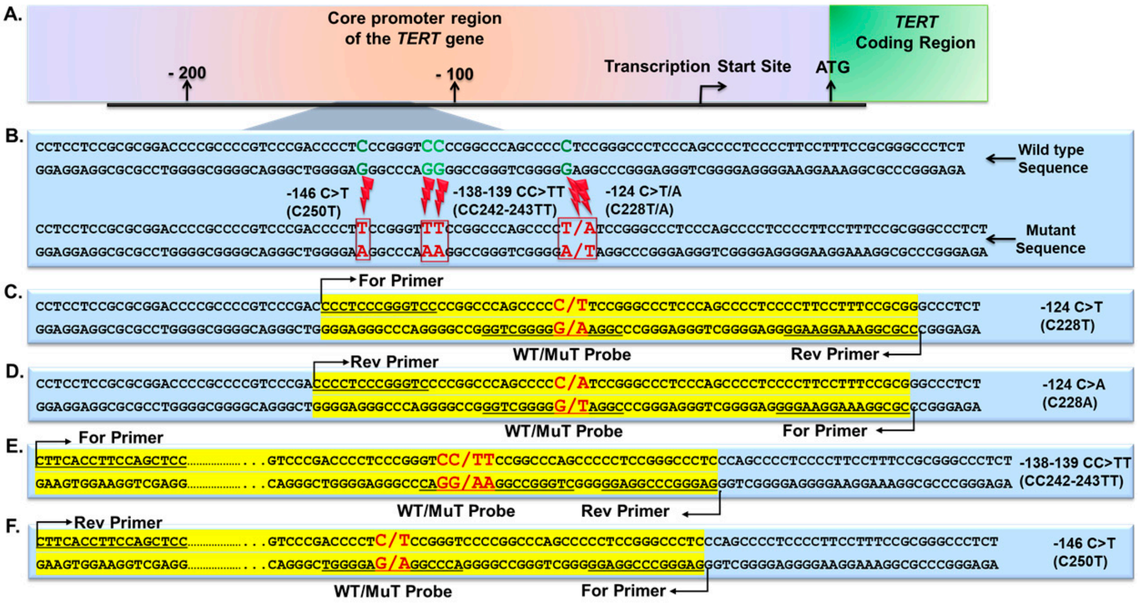

Figure S1. *TERT* core promoter region with the primer and probe sets designed for each of the *TERT* promoter mutation assays. The panel A shows the core promoter region and the ATG start site for the *TERT* gene. The panel B shows the position of the common and rare mutations (both wildtype and mutated sequences are shown here). The hotspot mutations (−124C/T or C228T and -146C/T or C250T) along with the rare mutations (−124C/A or C228A and −138-139CC/TT or CC242-243TT) mutations are detected using separate primer and probe sets for each of the mutations as shown in Panel C to F. The −124, −138, −139 and −146 positions refer to the upstream of the ATG start site, hence the '−'(minus) sign. The 228, 242, 243 and 250 positions are according to the genomic coordinates for the respective mutation positions as per the GRCh37/hg19 Human Genome Assembly (27 February 2009 release). For example, the C228T mutation is located at the chr5:1295228 position of the GRCh37/hg19 Human Genome Assembly. The PCR products for each assay are highlighted in yellow and the positions for the primer and probes are underlined.

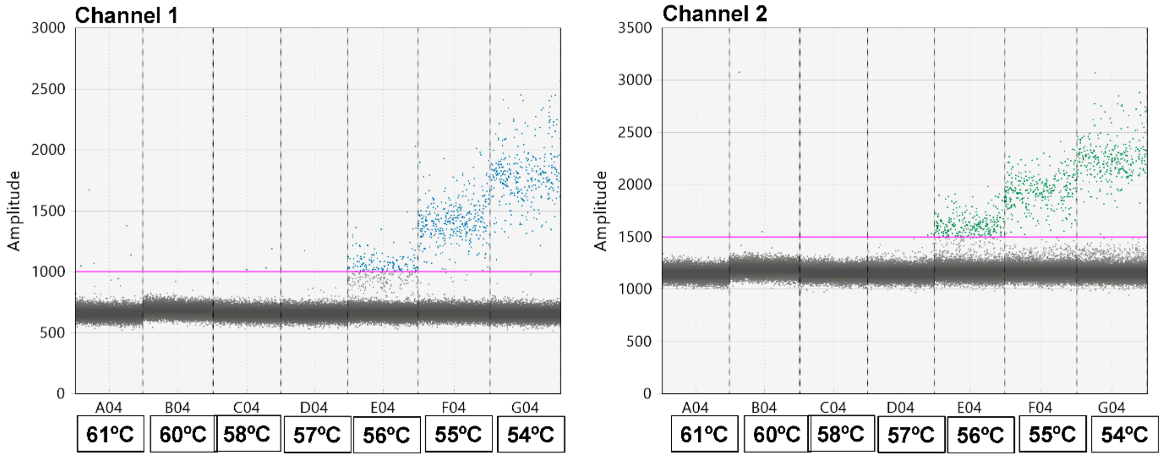

Figure S2. 1D amplitude plot for Channel 1 (respective to mutated probe) and Channel2 (respective for wildtype probe) for the C228T assay at different annealing temperatures. 54 °C shows a clear separation of clusters in both the channels.

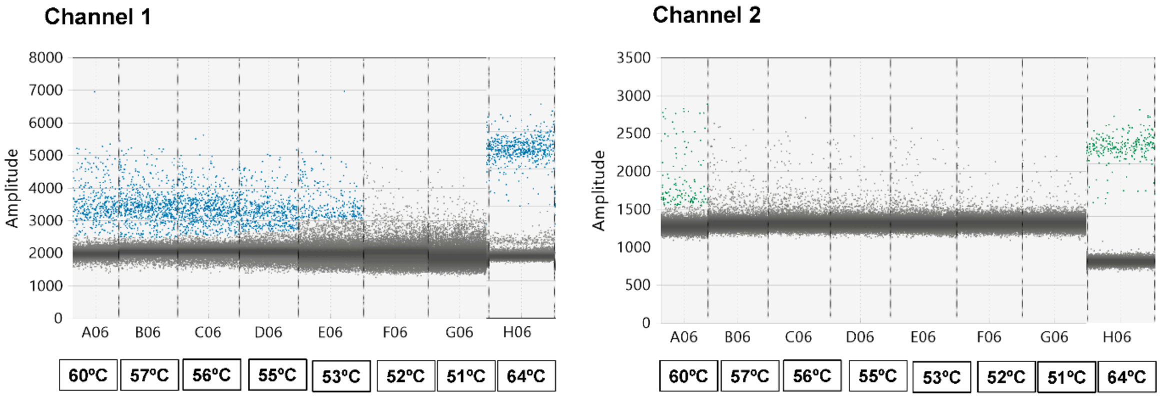

Figure S3. 1D amplitude plot for Channel1 (respective to mutated probe) and Channel2 (respective for wildtype probe) for the C250T assay at different annealing temperatures. 64 °C shows a clear separation of clusters in both the channels.

Table S1. Determination of Limit of Detection for the C228T mutations using cell line DNA.

Amount of Template DNA for ddPCR (ng)	Dilution Factor from HepG2 DNA (C228T Mutated)	WT Droplets	Total Mutated Droplets	ddPCR-MAF
5	None (100% HepG2)	109	112	50.6787
5	2	664	118	15.0895
5	5	882	55	5.8698
5	10	1074	35	3.1560
**5**	**20**	**971**	**6**	**0.6141**
5	*50*	*1028*	*5*	*0.4840*
5	*100*	*1162*	*3*	*0.2575*
5	*200*	*1030*	*4*	*0.3868*
5	*500*	*1009*	*0*	*0.0000*
5	*1000*	*999*	*2*	*0.1998*
5	*2000*	*1141*	*4*	*0.3493*
5	*5000*	*995*	*2*	*0.2006*
5	*10000*	*916*	*0*	*0.0000*
5	*None-100% wild-type*	*1336*	*3*	*0.2240*
10	None (100% HepG2)	299	326	52.16
10	2	429	209	32.76
10	5	202	49	19.52
10	10	424	63	12.94
10	20	394	27	6.41
**10**	**50**	**438**	**11**	**2.45**

10	*100*	*390*	*5*	*1.27*
10	*200*	*413*	*0*	*0.00*
10	*500*	*417*	*1*	*0.24*
10	*1000*	*498*	*2*	*0.40*
10	*2000*	*482*	*0*	*0.00*
10	*5000*	*604*	*1*	*0.17*
10	*10000*	*549*	*1*	*0.18*
10	*None-100% wild-type*	*4999*	*1*	*0.02*
20	None (100% HepG2)	1034	1130	52.22
20	2	1086	585	35.01
20	5	2190	180	7.59
20	10	984	119	10.79
20	20	2494	56	2.20
20	50	2615	20	0.76
20	100	2404	12	0.50
20	200	1020	7	0.68
**20**	**500**	**1005**	**7**	**0.69**
20	*1000*	*2642*	*0*	*0.00*
20	*2000*	*2886*	*1*	*0.03*
20	*5000*	*2391*	*1*	*0.04*
20	*10000*	*2650*	*1*	*0.04*
20	*None-100% wild-type*	*9342*	*3*	*0.03*
40	None (100% HepG2)	2297	2305	50.09
40	2	3445	930	21.26
40	5	5280	436	7.63
40	10	5029	204	3.90
40	20	5381	91	1.66
40	50	5948	37	0.62
40	100	5402	16	0.30
**40**	**200**	**6525**	**12**	**0.18**
40	*500*	*5946*	*3*	*0.05*
40	*1000*	*6095*	*4*	*0.07*
40	*2000*	*7785*	*7*	*0.09*
40	*5000*	*6520*	*1*	*0.02*
40	*10000*	*7587*	*0*	*0.00*
40	*None-100% wild-type*	*6140*	*1*	*0.02*

The threshold point for limit of detection is marked as bold and the dilutions that were below the threshold of the limit of detection are italicized.

Table S2. Determination of Limit of Detection for the C250T mutations using cell line DNA.

Amount of Template DNA for ddPCR (ng)	Proportion of A375 DNA (C250T Mutated)	WT Droplets	Total Mutated Droplets	ddPCR-MAF
5	None (100% A375)	326	739	69.39
5	2	676	339	33.40
5	5	1023	143	12.26
5	10	1168	87	6.93
5	20	1356	47	3.35
**5**	**50**	**1049**	**21**	**1.96**
5	*100*	*1158*	*4*	*0.34*
5	*200*	*1040*	*3*	*0.29*
5	*500*	*1922*	*5*	*0.26*
5	*1000*	*1107*	*3*	*0.27*
5	*2000*	*1176*	*1*	*0.08*
5	*5000*	*1153*	*1*	*0.09*
5	*10000*	*1116*	*0*	*0.00*
5	*None-100% wild-type*	*1662*	*2*	*0.12*
10	None (100% HepG2)	668	1513	69.37
10	2	1516	780	33.97
10	5	1513	244	13.89
10	10	1940	139	6.69
10	20	2309	70	2.94
10	50	2013	33	1.61

10	100	2353	27	1.13
**10**	**200**	**2108**	**9**	**0.43**
10	*500*	*2061*	*2*	*0.10*
10	*1000*	*2147*	*4*	*0.19*
10	*2000*	*1187*	*0*	*0.00*
10	*5000*	*2173*	*2*	*0.09*
10	*10000*	*1336*	*0*	*0.00*
10	*None-100% wild-type*	*2674*	*1*	*0.04*
20	None (100% A375)	648	1487	69.65
20	2	1253	731	36.84
20	5	1518	229	13.11
20	10	3516	147	4.01
20	20	2025	83	3.94
20	50	1785	25	1.38
20	100	1757	10	0.57
**20**	**200**	**2183**	**9**	**0.41**
20	*500*	*2659*	*3*	*0.11*
20	*1000*	*2349*	*2*	*0.09*
20	*2000*	*2477*	*3*	*0.12*
20	*5000*	*NA*	*NA*	*NA*
20	*10000*	*2198*	*2*	*0.09*
20	*None-100% wild-type*	*2598*	*1*	*0.04*
40	None (100% A375)	1632	4409	72.98
40	2	3821	2451	39.08
40	5	4964	971	16.36
40	10	5059	415	7.58
40	20	5408	248	4.38
40	50	5704	100	1.72
40	100	6197	49	0.78
40	200	7478	27	0.36
**40**	**500**	**6737**	**12**	**0.18**
40	*1000*	*7156*	*5*	*0.07*
40	*2000*	*7157*	*2*	*0.03*
40	*5000*	*6956*	*2*	*0.03*
40	*10000*	*6982*	*2*	*0.03*
40	*None-100% wild-type*	*7085*	*1*	*0.01*

The threshold point for limit of detection is marked as bold and the dilutions that were below the threshold of the limit of detection are italicized.

Table S3. Performance of urinary *TERT* promoter mutations in detecting UC by ddPCR based method.

**Cohort**	**DIAGURO Cohort**			**PORTO cohort**
**Sample Type**	**US cfDNA or UP DNA**	**US cfDNA**	**UP DNA**	**UP DNA**
**Numbers of Samples**	**(*N* = 187)**	**(*N* = 187)**	**(*N* = 187)**	**(*N* = 100)**
C228T or C250T	-	-	-	-
True Positive-no	79	66	77	33
True Negative-no	85	83	84	50
False positive-no	7	03	6	0
False negative-no	10	9	11	16
No data–no$	6	26	9	1
Sensitivity (95% CI)-%	86.8 (80.3–94.5)	88.0 (78.4–94.4)	87.5 (78.7–93.6)	67.4 (52.5–80.1)
Specificity (95% CI)-%	92.4 (85.0–96.9)	96.5(90.1–99.3)	93.3 (86.1–97.5)	100.0 (92.9–100.0)
Accuracy (95% CI)-%	91.3 (86.2–95.0)	94.0 (89.1–97.1)	91.6 (86.5–95.2)	90.2 (82.6–95.3)

US: Urine Supernatant; UP: Urine Pellet. $: Samples with either insufficient quantity of DNA to be screened by ddPCR or samples poor NGS or ddPCR data that could not be exploited.

Table S4. Concordance of TERT promoter mutations status in urinary DNA of the DIAGURO and IPO-PORTO case-control studies.

**US cfDNA**	**ddPCR-MUT**	**ddPCR-WT**	**ddPCR-Failed**	**TOTAL**	Kappa 0.91

UroMuTERT-MUT	64	3	1	68	
UroMuTERT-WT	4	86	0	90	
UroMuTERT-Failed	1	5	-	6	
TOTAL	69	94	1	164	
**UP cellDNA**	**ddPCR-MUT**	**ddPCR-WT**	**ddPCR-Failed**	**TOTAL**	
UroMuTERT-MUT	111	2	1	114	
UroMuTERT-WT	6	159	0	165	Kappa 0.94
UroMuTERT-Failed	1	0	-	1	
TOTAL	118	161	1	280	

Table S5. Probes for detecting C228T and C250T mutations in the ddPCR assay.

Mutation Type	Primer/Probe	Sequence (5′ to 3′)	Fluorescent Dye and Quencher	PCR Product Size (bp)
TERT C228T	fw_primer	CCCTCCCGGGTCC	-	64
	rev_primer	CCGCGGAAAGGAAGG	-	
	wt_probe	CGGAgGGGGCTGG	HEX_IowaBlack	
	mut_probe	CCCGGAaGGGGCTG	FAM_IowaBlack	
TERT C250T	fw_primer	CTTCACCTTCCAGCTCC	-	88
	rev_primer	GAGGGCCCGGAGG	-	
	wt_probe	ACCCGGgAGGGGT	HEX_IowaBlack	
	mut_probe	CCCGGaAGGGGTCG	FAM_IowaBlack	
TERT C228A	fw_primer	CGCGGAAAGGAAGGG	-	64
	rev_primer	CCCCTCCCGGGTC	-	
	wt_probe	CGGAgGGGGCTGG	HEX_IowaBlack	
	mut_probe	CCCGGAtGGGGCTG	FAM_IowaBlack	
TERT CC242–243TT	fw_primer	GAGGGCCCGGAGG	-	88
	rev_primer	CTTCACCTTCCAGCTCC	-	
	wt_probe	CTGGGCCGGggAC	HEX_IowaBlack	
	mut_probe	CCGGaaACCCGGGA	FAM_IowaBlack	

Fw: Forward; rev: Reverse; wt: Wild-type; mut: Mutated, ddPCR probes containing either a 5′-FAM or 5′-HEX reporter dye and 3′ Iowa Black® Fluorescent quencher were HPLC purified.

Publisher's Note: MDPI stays neutral with regard to jurisdictional claims in published maps and institutional affiliations.

© 2020 International Agency for Research on Cancer (IARC); Licensee MDPI, Basel, Switzerland. This is an open access article distributed under the terms of the Creative Commons Attribution IGO License (CC BY) (http://creativecommons.org/licenses/by/3.0/igo/legalcode), which permits unrestricted use, distribution, and reproduction in any medium, provided the original work is properly cited. In any reproduction of this article there should not be any suggestion that IARC or this article endorse any specific organisation or products. The use of the IARC logo is not permitted. This notice should be preserved along with the article's original URL.
